# *Campylobacter jejuni* Induces Acute Enterocolitis in Gnotobiotic IL-10^−/−^ Mice via Toll-Like-Receptor-2 and -4 Signaling

**DOI:** 10.1371/journal.pone.0040761

**Published:** 2012-07-10

**Authors:** Lea-Maxie Haag, André Fischer, Bettina Otto, Rita Plickert, Anja A. Kühl, Ulf B. Göbel, Stefan Bereswill, Markus M. Heimesaat

**Affiliations:** 1 Department of Microbiology and Hygiene, Charité - University Medicine Berlin, Berlin, Germany; 2 Department of Internal Medicine, Rheumatology and Clinical Immunology/Research Center ImmunoSciences (RCIS), Charité - University Medicine Berlin, Berlin, Germany; Biological Research Centre of the Hungarian Academy of Sciences, Hungary

## Abstract

**Background:**

*Campylobacter jejuni* is a leading cause of foodborne bacterial enterocolitis worldwide. Investigation of immunopathology is hampered by a lack of suitable vertebrate models. We have recently shown that gnotobiotic mice as well as conventional IL-10^−/−^ animals are susceptible to *C. jejuni* infection and develop intestinal immune responses. However, clinical symptoms of *C. jejuni* infection were rather subtle and did not reflect acute bloody diarrhea seen in human campylobacteriosis.

**Methodology/Principal Findings:**

In order to overcome these limitations we generated gnotobiotic IL-10^−/−^ mice by quintuple antibiotic treatment starting right after weaning. The early treatment was essential to prevent these animals from chronic colitis. Following oral infection *C. jejuni* colonized the gastrointestinal tract at high levels and induced acute enterocolitis within 7 days as indicated by bloody diarrhea and pronounced histopathological changes of the colonic mucosa. Immunopathology was further characterized by increased numbers of apoptotic cells, regulatory T-cells, T- and B-lymphocytes as well as elevated TNF-α, IFN-γ, and MCP-1 concentrations in the inflamed colon. The induction of enterocolitis was specific for *C. jejuni* given that control animals infected with a commensal *E. coli* strain did not display any signs of disease. Most strikingly, intestinal immunopathology was ameliorated in mice lacking Toll-like-receptors-2 or -4 indicating that *C. jejuni* lipoproteins and lipooligosaccharide are essential for induction and progression of immunopathology.

**Conclusion/Significance:**

Gnotobiotic IL-10^−/−^ mice develop acute enterocolitis following *C. jejuni* infection mimicking severe episodes of human campylobacteriosis and are thus well suited to further dissect mechanisms underlying *Campylobacter* infections *in vivo*.

## Introduction

*Campylobacter (C.) jejuni* is a leading cause of bacterial-induced enteritis worldwide. The zoonotic pathogen forms part of the commensal flora in many wild and domestic animals. Broiler chicks are primarily involved in transmitting this pathogen to humans [1], [2], [3]. Clinical symptoms of human campylobacteriosis vary from mild malaise to severe ulcerative enterocolitis requiring hospitalization in severely (immuno-) compromized patients. In most cases, however, infection of humans is self-limiting [4]. In the acute stage of *C. jejuni* induced enterocolitis, infected patients present with bloody diarrhea, abdominal cramps and fever. Histological examination of affected intestinal tissue sites reveals crypt abscesses, ulcerations, and elevated immune cell numbers in the colon *in situ* [5], [6], [7], [8]. In small subgroups of patients the acute phase is followed by serious sequelae, including Guilliane-Barré-Syndrome, Miller-Fischer-Syndrome and reactive arthritis [9], [10]. Despite the high prevalence of *C. jejuni* induced disease and its distinctive socioeconomic impact, molecular and cellular events leading to campylobacteriosis are still poorly understood [11]. A very recent review article from Ó Cróinin and Backert highlighted various pathogenicity factors and host cell determinants proposed to be involved in establishing *C. jejuni* infection and triggering disease [12]. Eventhough colonization factors of *C. jejuni* are well known, paucity of understanding molecular mechanisms underlying *C. jejuni* induced immunopathology is mainly due to the scarcity of suitable experimental *in vivo* models of human infection [13]. Mice are generally highly convenient for studying pathogenicity of bacteria and immunopathology. Most murine *C. jejuni* infection models analyzed so far suffer from sporadic colonization and the lack of intestinal immunopathology. Mice harboring a conventional commensal gut microbiota can often not be stably colonized by *C. jejuni* [6], [13]. Eventhough this phenomenon of “colonization resistance” in immunocompetent mice has been known for decades the mechanistic basis of this physiological barrier and how it can be overcome by commensal or pathogenic bacteria is still incompletely defined [14], [15], [16]. Some studies with isolator raised germfree mice underlined the substantial role of the murine microbiota in colonization resistance against *C. jejuni*. Following oral infection, *C. jejuni* colonized the entire gastrointestinal tract of these animals and induced clinical signs of disease including granulocyte infiltrates and bloody diarrhea, as well as humoral immune responses [17], [18], [19]. Despite the increased susceptibility to *C. jejuni* infection, germfree mice do not represent a suitable experimental model of *C. jejuni* infections due to the absence of an intact immune system [20], [21]. More recent investigations of our group revealed that colonization resistance is mainly caused by the host-specific intestinal microbiota composition and can be overcome under certain circumstances: Following peroral *C. jejuni* infection gnotobiotic wildtype mice, gnotobiotic mice reconstituted with a human microbiota and animals fed a Western style cafeteria diet could be readily colonized by the pathogen and displayed pro-inflammatory immune responses within the colon [22], [23]. In addition, acute as well as chronic inflammation within the small or large intestine has been shown to facilitate *C. jejuni* infection due to higher intestinal enterobacteria loads such as *E. coli*. In a very recent study we could clearly demonstrate that artificially elevating the intestinal *E. coli* burden by feeding live bacteria to adult mice harboring a conventional gut microbiota (and thus rendering them resistant to *C. jejuni* infection) abolished colonization resistance and *C. jejuni* infection induced pro-inflammatory immune responses within the infected gut [24]. However, severe clinical symptoms of acute enterocolitis such as bloody diarrhea in human campylobacteriosis were missing in so far generated animal models. Very recently we demonstrated that infection of 3-weeks-old infant mice right after weaning, but not adult 3-months-old animals harboring an age-dependent conventional intestinal microbiota developed acute enterocolitis within 6 days p.i. as indicated by bloody diarrhea, colonic shortening and increased apoptotic cell numbers in the colon mucosa. Similar to human campylobacteriosis, disease was self-limited and resolved within two weeks [25].

We here demonstrate that following *C. jejuni* infection gnotobiotic IL-10^−/−^ mice develop acute enterocolitis as in human campylobacteriosis. Antibiotic treatment right after weaning prevented IL-10^−/−^ mice from development of chronic colitis which usually becomes overt between 3 to 6 months of age, depending on housing conditions and the specific gut microbiota composition [26]. Toll like receptors (TLR) mediate essential signaling pathways involved in innate and adaptive host responses to commensal and pathogenic bacteria. Bacterial lipoproteins (LP) and lipooligosaccharides (LOS) are recognized by TLRs 2 and -4, respectively [27]. Detailed investigation of the interaction between *C. jejuni* and individual TLRs *in vivo* and in the intestines *in situ* are rather scarce. In different human, murine and avian cell lines *C. jejuni* was shown to activate TLR-2, TLR-4 and TLR-9 via MyD88 [28], [29], [30], [31], [32]. We have recently demonstrated that TLR-4 and -9 signaling are essential for induction of intestinal inflammation by *C. jejuni* in gnotobiotic mice which develop rather subtle symptoms [22]. The crucial role of TLRs and defined bacterial molecules in *C. jejuni* mediated intestinal immunopathology was now independently confirmed for lipoproteins and lipooligosaccharide in gnotobiotic IL-10^−/−^ mice lacking TLRs 2 or 4.

## Results

### Acute Enterocolitis in *C. jejuni* Infected Gnotobiotic IL-10^−/−^ Mice

We have recently shown that 5 to 6 months old IL-10^−/−^ mice harboring a conventional intestinal microbiota and suffering from chronic colitis could be stably infected with *C. jejuni* at intermediate levels in approximately 70% of cases, but symptoms were rather subtle and, hence, ”classical” clinical signs of severe human camplyobacteriosis such as bloody diarrhea were missing. In this study, we eradicated the chronic colitogenic stimulus in IL-10^−/−^ mice by quintuple antibiotic treatment for 4–5 months starting right after weaning. These gnotobiotic IL-10^−/−^ mice were then perorally challenged either with *C. jejuni* B2 or a commensal *E. coli* strain lacking any relevant pathogenicity factors such as *stx* 1 and 2, *catA*, *hlyA*, *cspA*, *katP*, and *astA* which had been isolated from the colonic lumen of a conventional wildtype mouse [24] (see Materials and Methods). *C. jejuni* reference strain B2 originates from a patient with severe enteritis, is invasive *in vitro* and has been extensively investigated in earlier studies [33]. Naïve, uninfected gnotobiotic IL-10^−/−^ mice without any signs of intestinal inflammation served as negative controls.

**Figure 1:**
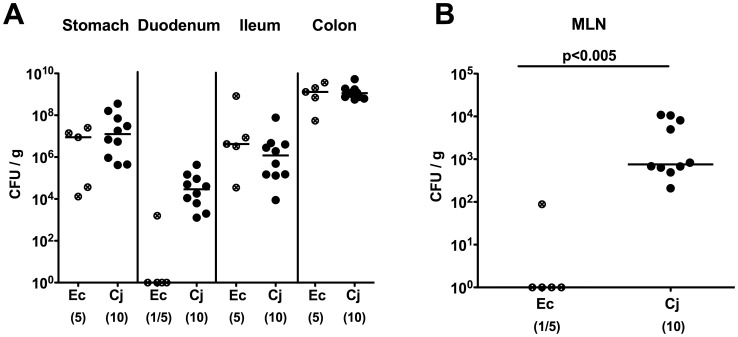
*C. jejuni* B2 colonization and translocation in gnotobiotic IL-10^−/−^ mice. Gnotobiotic IL-10^−/−^ mice were generated by antibiotic treatment and orally infected either with *C. jejuni* B2 (solid circles) or an apathogenic *E. coli* strain (crossed circles) derived from the commensal gut microbiota of conventional colonized wildtype mice as described in Methods. *C. jejuni* B2 and *E. coli* loads were determined in luminal samples taken from different compartments of the gastrointestinal tract (**A**) and mesenteric lymphnodes (MLN) (**B**) at day 7 p.i. (CFU, colony forming units). Numbers of animals harboring the respective bacterial species out of the total number of analyzed animals are given in parentheses. Medians (black bars) and significance levels (*P*-values) determined by Mann-Whitney-U test are indicated. Data shown are representative for three independent experiments.

**Figure 2:**
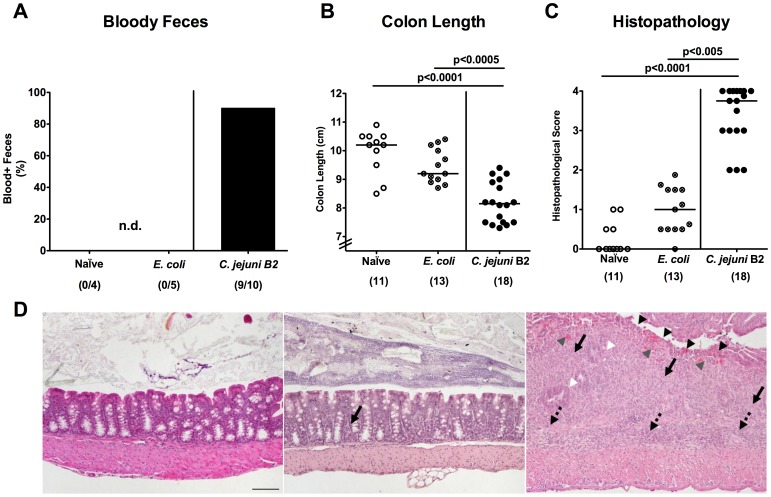
*C. jejuni* induces acute enterocolitis in gnotobiotic IL-10^−/−^ mice. Gnotobiotic IL-10^−/−^ mice were generated by antibiotic treatment and orally infected either with *C. jejuni* B2 (solid circles; black bar) or a commensal *E. coli* strain (crossed circles) derived from the commensal gut microbiota of conventional colonized wildtype mice as described in Methods. Uninfected gnotobiotic IL-10^−/−^ mice served as negative controls (open circles). (**A**) Relative rate of blood-positive stool samples of infected IL-10^−/−^ and uninfected control mice were determined by haemoccult test at day 7 p.i. (see Methods). Numbers of animals with haemcoocult positive stool samples out of the total number of analyzed animals are given in parentheses (n.d., not detectable). (**B**) Absolute colon lengths and (**C**) histopathological changes applying a standardized histopathological score in HE-stained colonic paraffin sections were determined at day 7 p.i., illustrated by (**D**) representative photomicrographs out of three independent experiments (100x magnification, scale bar 100 µm) derived from naïve (left), *E. coli*- (middle) and *C. jejuni* B2- (right) infected animals. Solid arrows indicate mucosal, dotted arrows submucosal infiltrates, black arrow heads ulcerations, white arrow heads loss of goblet cells, and gray arrow heads mucosal bleeding. Numbers of analyzed animals are given in parentheses. Medians (black bars) and significance levels (*P*-values) determined by Mann-Whitney-U test are indicated. Data shown were pooled from at least three independent experiments.

Seven days following peroral infection *C. jejuni* B2 and *E. coli* stably colonized the gastrointestinal tract of gnotobiotic IL-10^−/−^ mice at comparably high levels of approximately 10^9^ colony forming units (CFU) per gram (**Fig. 1A**). Interestingly *C. jejuni* B2, but not *E. coli*, translocated to mesenteric lymphnodes draining the large intestine at day 7 p.i. (**Fig. 1B**). Strikingly, *C. jejuni* B2 infected animals were decisively compromized by wasting enterocolitis as indicated by diarrhea, occurrence of blood in 80% of fecal samples in infected mice (**Fig. 2A**) and a significant reduction of the mean colonic length by approximately 20% within 7 days p.i. (**Fig. 2B**). Of note, approximately 30% of *C. jejuni* B2 infected animals had deceased before necropsy between day 4 and 7 p.i. (not shown). *E. coli* infected mice, however, were virtually unaffected and clinically comparable to naïve controls (**Fig. 2**). Acute *C. jejuni* B2 induced enterocolitis was further confirmed by distinct histopathological changes of the colonic mucosa as indicated by significantly higher histopathological scores in *C. jejuni* B2 infected as compared to naive and *E. coli* infected gnotobiotic IL-10^−/−^ mice (**Fig. 2C**). Acute ulcerative colitis in diseased IL-10^−/−^ mice was characterized by ulcerations of and bleeding into the colonic mucosa as well as diffuse mucosal and submucosal infiltrates, and, in additon, by loss of goblet cells and crypt drop-outs at day 7 p.i. (**Fig. 2D**). Taken together, acute enterocolitis in gnotobiotic IL-10^−/−^ mice following *C. jejuni*, but not *E. coli* infection mimicks clinical symptoms of severe human campylobacteriosis and is specifically induced by the pathogen.

### Inflammatory Immune Responses in *C. jejuni* Infected Gnotobiotic IL-10^−/−^ Mice

**Figure 3:**
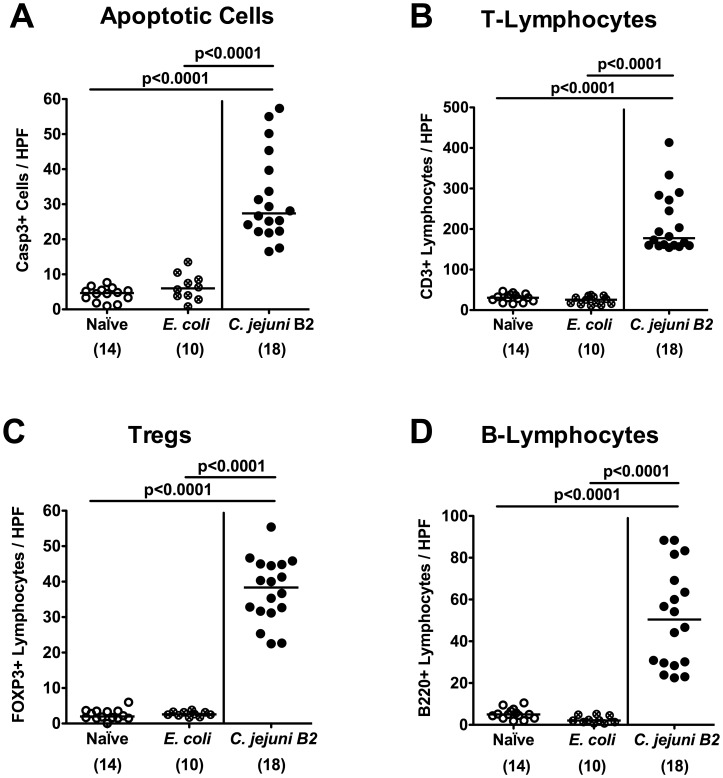
Inflammatory and immune cell responses following *C. jejuni* infection of gnotobiotic IL-10^−/−^ mice. Gnotobiotic IL-10^−/−^ mice were generated by antibiotic treatment and orally infected either with *C. jejuni* B2 (solid circles) or an apathogenic *E. coli* strain (crossed circles) derived from the commensal gut microbiota of conventional colonized wildtype mice as described in Methods. Uninfected gnotobiotic IL-10−/− mice served as negative controls (open circles). The average numbers of apoptotic cells (positive for caspase-3, panel **A**), T-lymphocytes (positive for CD3, panel **B**), regulatory T-cells (Tregs, positive for FOXP3, panel **C**) and B-lymphocytes (positive for B220, panel **D**) from at least six high power fields (HPF, 400x magnification) per animal were determined microscopically in immunohistochemically stained colon sections at day 7 p.i. Numbers of analyzed animals are given in parentheses. Means (black bars) and levels of significance (*P*-values) determined by the Student’s *t*-test are indicated. Data shown are pooled from at least three independent experiments.

**Figure 4:**
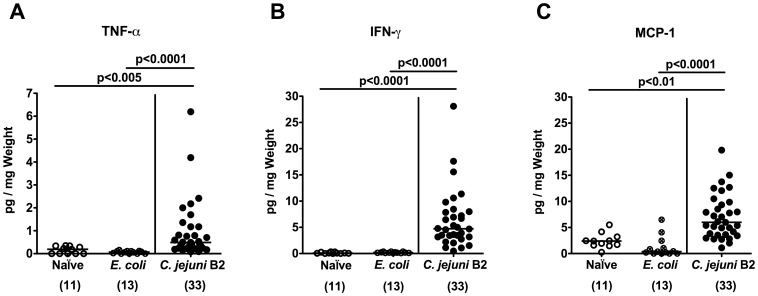
Pro-inflammatory cytokine responses in the colon of *C. jejuni* infected gnotiotic IL-10^−/−^ mice. Gnotobiotic IL-10^−/−^ mice were generated by antibiotic treatment and orally infected with either *C. jejuni* B2 (solid circles) or an apathogenic *E. coli* strain (crossed circles) derived from the commensal gut microbiota of conventional colonized wildtype mice as described in Methods. Uninfected gnotobiotic IL-10^−/−^ mice served as negative controls (open circles). Concentrations (pg per mg colon) of (**A**) TNF-α, (**B**) IFN-γ, and (**C**) MCP-1 were determined in supernatants of *ex vivo* colonic cultures at day 7 p.i. by cytometric bead array (CBA). Numbers of analyzed animals are given in parentheses. Means (black bars) and levels of significance (*P*-values) as compared to the respective group (determined by Student’s *t*-test) are indicated. Data shown are pooled from at least three independent experiments.

To further elucidate whether *C. jejuni* infection of gnotobiotic IL-10^−/−^ mice induced pro-inflammatory immune responses, we analyzed apoptotic and distinct immune cell populations in the colonic mucosa *in situ. C. jejuni* B2 infected gnotobiotic IL-10^−/−^ mice displayed multifold (between 5 to 15 times) higher numbers of apoptotic cells, T- and B-lymphocytes as well as regulatory T-cells in the colonic mucosa at day 7 p.i. as compared to *E. coli* infected and naive control animals (**Fig. 3**). The immune cell responses were accompanied by increased expression of pro-inflammatory cytokines and mediators in the colon. Levels of TNF-α, IFN-γ, and MCP-1 were significantly higher in *ex vivo* colonic biopsies derived from *C. jejuni* B2 infected mice at day 7 p.i. as compared to *E. coli* infected and naive controls (**Fig. 4**). Again, pro-inflammatory immune cell responses were absent in *E. coli* infected as well as in un-infected, naïve gnotobiotic IL-10^−/−^ mice (**Fig. 3** **,** **Fig. 4**).

### *C. jejuni* Colonization and Translocation in Gnotobiotic IL-10^−/−^ Mice Lacking TLR-2 or TLR-4

**Figure 5:**
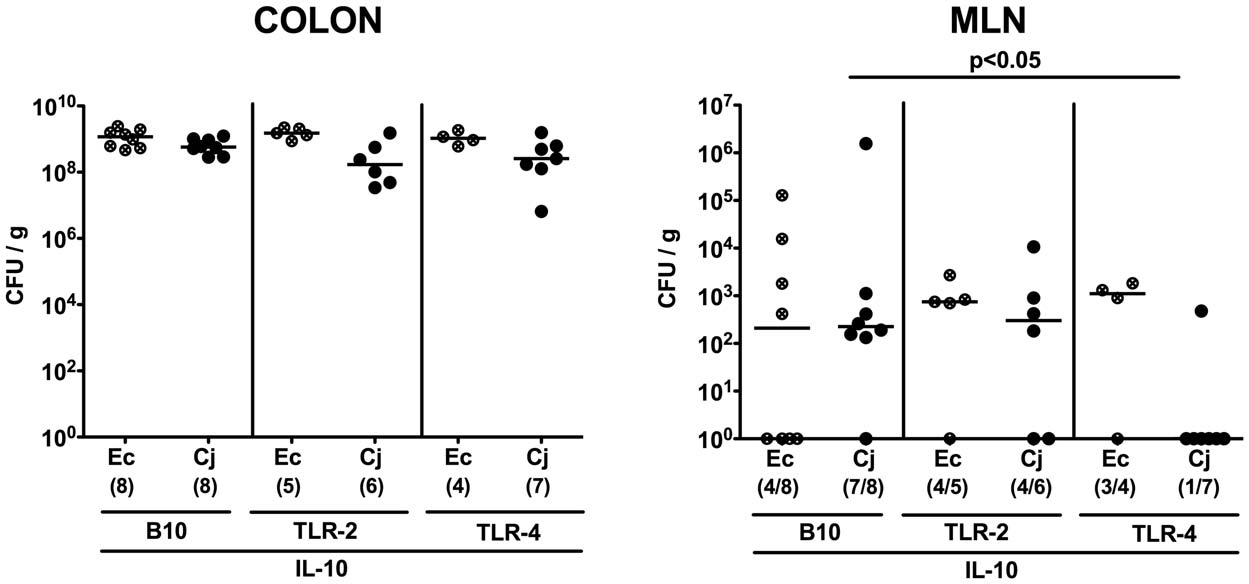
TLR-dependent *C. jejuni* colonization and translocation in gnotobiotic IL-10^−/−^ mice. Gnotobiotic IL-10^−/−^ (B10 background) as well as TLR-2^−/−^ IL-10^−/−^ and TLR-4^−/−^ IL-10^−/−^ mice were generated by antibiotic treatment and orally infected either with *C. jejuni* B2 (C*j*; solid symbols) or a commensal *E. coli* strain (Ec; crossed symbols) as described in Methods. *C. jejuni* B2 and *E. coli* loads were determined in luminal colonic samples (**A**) and mensenteric lymphnodes (MLN) (**B**) at day 7 p.i. (CFU, colony forming units). Numbers of animals harboring the respective bacterial species out of the total number of analyzed animals are given in parentheses. Medians (black bars) and significance levels (*P*-values) determined by Mann-Whitney-U test are indicated. Data shown are representative for three independent experiments.

To gain further insights into the immunopathological mechanisms underlying acute *C. jejuni* induced enterocolitis *in vivo* we crossed IL-10^−/−^ mice with animals deficient in TLR-2 or -4 recognizing bacterial lipoproteins and lipooligosaccharide, respectively (all in B10 background, see Methods), and generated gnotobiotic double-deficient mice by quintuple antibiotic treatment for 4–5 months starting right after weaning. In the naïve condition, neither gnotobiotic TLR-2 IL-10 nor TLR-4 IL-10 double deficient mice displayed any clinical or histopathological signs of intestinal inflammation at all (not shown). At day 7 following oral *C. jejuni* B2 or *E. coli* infection gnotobiotic mice of either genotype harbored comparable loads of the respective bacterial species in their colon lumen (**Fig. 5A**). Thus, neither TLR-2 nor TLR-4 deficiency affected colonization capacity of *C. jejuni* B2 in gnotobiotic IL-10^−/−^ animals.

Whereas in 87.5% and 66.7% of IL-10^−/−^ and TLR-2 IL-10 double deficient mice, respectively, live *C. jejuni* B2 were detected in MLNs, only in 14.3% of TLR-4 IL-10 double deficient animals the pathogen could be cultured from MLNs taken from at day 7 p.i. (**Fig. 5B**). Interestingly, no bacterial translocation from the intestinal tract to other extra-intestinal organs such as spleen, liver, kidney or cardiac blood occurred at day 7 p.i., irrespective of the genotype of infected mice (not shown).

### Acute Enterocolitis in *C. jejuni* Infected Gnotobiotic IL-10^−/−^ Mice Lacking TLR-2 or TLR-4

**Figure 6:**
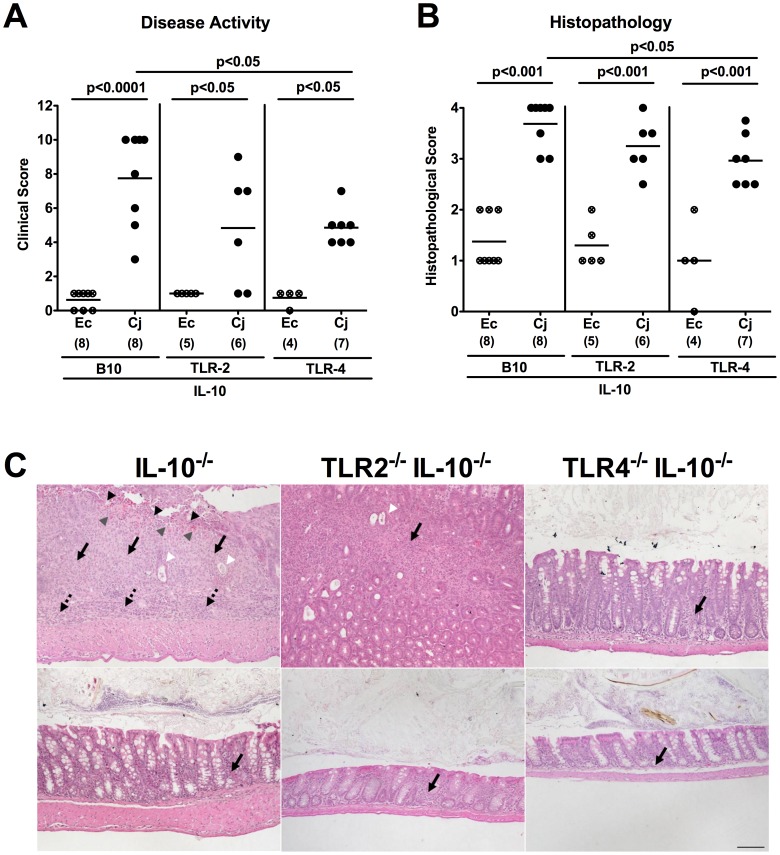
*C. jejuni* induces TLR-dependent acute enterocolitis in gnotobiotic IL-10^−/−^ mice. Gnotobiotic IL-10^−/−^ (B10 background) as well as TLR-2^−/−^ IL-10^−/−^ and TLR-4^−/−^ IL-10^−/−^ mice were generated by antibiotic treatment and orally infected either with *C. jejuni* B2 (Cj; solid symbols) or a commensal *E. coli* strain (Ec; crossed symbols) derived from the commensal gut microbiota of conventional colonized wildtype mice as described in Methods. (**A**) Disease activity of infected mice and (**B**) histopathological changes in HE-stained colonic paraffin sections applying a standardized clinical and histopathological score, respectively, were determined at day 7 p.i., illustrated by (**C**) representative photomicrographs out of three independent experiments (100x magnification, scale bar 100 µm) derived from *C. jejuni* B2- (upper panel) and *E. coli*- (lower panel) infected IL-10^−/−^ (left panel), TLR-2^−/−^ IL-10^−/−^ (middle panel), and TLR-4^−/−^ IL-10^−/−^ (right panel) mice. Solid arrows indicate mucosal, dotted arrows submucosal iinfiltrates, black arrow heads ulcerations, white arrow heads crypt drop-out, and gray arrow heads mucosal bleeding. Numbers of analyzed animals are given in parentheses. Medians (black bars) and significance levels (*P*-values) determined by Mann-Whitney-U test are indicated. Data shown are representative for three independent experiments.

At day 7 p.i., *C. jejuni* B2 infected TLR-4 IL-10 double deficient mice exhibited less severe acute enterocolitis when compared to IL-10^−/−^ mice as indicated by significantly lower clinical scores assessing clinical condition, stool consistency and occurrence of blood in the stool. Additionally, histopathological changes of colonic mucosa in infected TLR-4 IL-10 double deficient mice were less distinct compared to IL-10^−/−^ controls whereas in IL-10^−/−^ animals lacking TLR-2 only a trend towards lower clinical and histopathological scores could be observed. This trend, however, did not reach statistical significance due to a relatively high standard deviation in TLR-2 IL-10 double deficient mice (**Fig. 6A-C**). Thus, TLR-4 and less distinctly TLR-2 mediate acute enterocolitis in gnotobiotic IL-10^−/−^ mice following *C. jejuni* B2 infection.

### Inflammatory Immune Responses in *C. jejuni* Infected Gnotobiotic IL-10^−/−^ Mice Lacking TLR-2 or TLR-4

**Figure 7:**
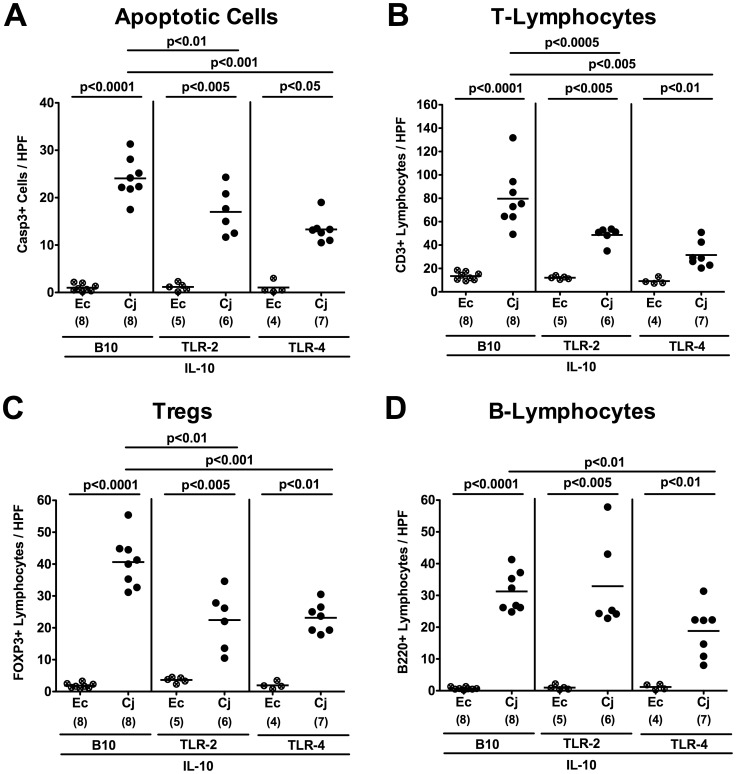
TLR-dependent inflammatory and immune cell responses following *C. jejuni* infection of gnotobiotic IL-10^−/−^ mice. Gnotobiotic IL-10^−/−^ (B10 background) as well as TLR-2^−/−^ IL-10^−/−^ and TLR-4^−/−^ IL-10^−/−^ mice were generated by antibiotic treatment and orally infected either with *C. jejuni* B2 (Cj; solid symbols) or a commensal *E. coli* strain (Ec; crossed symbols) as described in Methods. The average numbers of apoptotic cells (positive for caspase-3, panel **A**), T-lymphocytes (positive for CD3, panel **B**), regulatory T-cells (Tregs, positive for FOXP3, panel **C**) and B-lymphocytes (positive for B220, panel D) from at least six high power fields (HPF, 400x magnification) per animal were determined microscopically in immunohistochemically stained colon sections at day 7 p.i. Numbers of analyzed animals are given in parentheses. Means (black bars) and levels of significance (*P*-values) determined by the Student’s *t*-test are indicated. Data shown are representative for three independent experiments.

In humans, *C. jejuni* induces the recruitment of pro-inflammatory immune cell populations to sites of inflammation in the colon [7], [8]. Given that quantitative assessment of apoptotic and infiltrating immune cells in the colonic mucosa is more sensitive than applying the used histopathological score, we performed *in situ* immunohistochemical staining of paraffin colonic sections. At day 7 p.i., *C. jejuni* infected IL-10^−/−^ mice displayed two-fold higher numbers of apoptotic cells, T-lymphocytes and Tregs as compared to IL-10^−/−^ mice additionally lacking TLR-2 or TLR-4 (**Fig. 7A-C**). Furthermore, TLR-4 IL-10 double deficient mice exhibited lower B-lymphocyte numbers in their colonic mucosa at day 7 p.i. as compared to IL-10^−/−^ controls (**Fig. 7D**).

**Figure 8:**
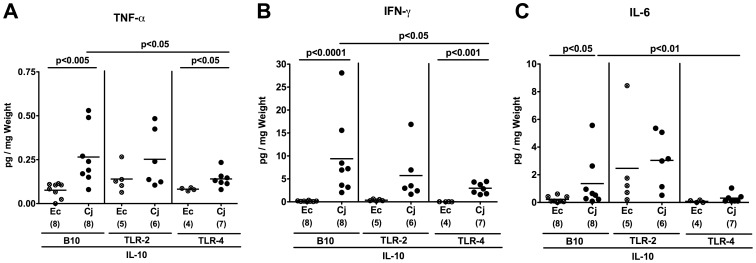
TLR-dependent pro-inflammatory cytokine responses in the colon of *C. jejuni* infected gnotobiotic IL-10^−/−^ mice. Gnotobiotic IL-10^−/−^ (B10 background) as well as TLR-2^−/−^ IL-10^−/−^ and TLR-4^−/−^ IL-10^−/−^ mice were generated by antibiotic treatment and orally infected either with *C. jejuni* B2 (Cj; solid symbols) or a commensal *E. coli* strain (Ec; crossed symbols) as described in Methods. Concentrations (pg per mg colon) of (**A**) TNF-α, (**B**) IFN-γ, and (**C**) IL-6 were determined in supernatants of *ex vivo* colon cultures at day 7 p.i. by cytometric bead array (CBA). Numbers of analyzed animals are given in parentheses. Means (black bars) and levels of significance (*P*-values) as compared to the respective group (determined by Student’s *t*-test) are indicated. Data shown are representative for three independent experiments.

The less distinct apoptotic mucosal changes and colonic immune cell infiltration observed in TLR-4 IL-10 double deficient versus IL-10^−/−^ mice were accompanied by significantly less secretion of pro-inflammatory cytokines such as TNF-α, IFN-γ, and IL-6 in *ex vivo* colonic biopsies taken at day 7 p.i. (**Fig. 8**). Again, oral *E. coli* infection did not induce intestinal inflammation in gnotobiotic animals irrespective of the investigated genotypes (**Fig. 7** **,** **8**).

Taken together, in this study we present a murine *C. jejuni* induced acute intestinal inflammation in gnotobiotic IL-10 deficient mice characterized by acute enterocolitis leading to death in some uneventful cases thus mimicking severe episodes of human camplyobacteriosis as seen in immunocompromized patients. Furthermore, TLR-4 signaling of *C. jejuni* lipooligosaccharide, and to a less distinct extent, TLR-2 signaling of lipoprotein mediates acute immunopathology following *C jejuni* infection. The presented model proves useful for further dissecting the immunopathological mechanisms underlying *Campylobacter* infections *in vivo* and to elucidate the interplay between intestinal pathogens, the commensal intestinal microbiota and the innate as well as adaptive immune system of the host.

## Discussion

Despite the high prevalence of campylobacteriosis in humans and its associated socioeconomic burden molecular mechanisms underlying immunopathology of *C. jejuni* infection in the host are still incompletely understood. This is mainly due to the strong colonization resistance of mice leading to sporadic colonization (if at all) and the absence of disease defining clinical manifestations. Previous studies of our group underlined the role of the host microbiota and its composition in maintaining colonization resistance. Using gnotobiotic wildtype mice in which the microbiota had been completely eradicated by antibiotic treatment or gnotobiotic mice reconstituted with a human microbiota oral *C. jejuni* infection resulted in stable gastrointestinal colonization and pro-inflammatory immune responses in the colon [22], [23]. Furthermore, we could demonstrate that conventional mice suffering from chronic colitis due to IL-10 deficiency could be stably infected with *C. jejuni* at intermediate levels for two weeks [24]. Severe clinical symptoms of human campylobacteriosis such as acute ulcerative enterocolitis seen in immunocompromized patients, however, were missing in these animal models. Given that firstly, *C. jejuni* infection of gnotobiotic mice resulted in stable colonization and, secondly, production of IL-10 and other anti-inflammatory mediators are involved in limiting *C. jejuni* induced immunopathology, we generated IL-10^−/−^ mice lacking any intestinal bacteria following quintuple antibiotic treatment for 5 months. One key feature of the novel infection model presented here is the very early beginning of antibiotic treatment in IL-10^−/−^ mice: Eradicating any commensal bacteria right after weaning abolished the main trigger for inducing and perpetuating chronic colitis in IL-10^−/−^ animals. Of note, following antibiotic treatment gnotobiotic IL-10^−/−^ mice displayed neither any clinical nor immunopathological signs of large intestinal inflammation prior infection. Following *C. jejuni* infection, however, gnotobiotic IL-10^−/−^ mice developed acute enterocolitis leading to death within one week in severe cases. Acute inflammation was characterized by rectal bleeding, histopathological signs of ulcerative colitis, increased numbers of apoptotic cells and increased recruitment of innate immune as well as effector cells in the colonic mucosa. In addition, an increased secretion of pro-inflammatory cytokines such as TNF-α, IFN-γ and MCP-1 was observed in *ex vivo* colonic cultures taken from *C. jejuni* infected mice. These findings are well in line with earlier studies using epithelial cell lines and bone marrow-derived dendritic cells. *C. jejuni* was shown to up-regulate chemokines involved in inflammatory responses such as MCP-1 and induced high level secretion of TNF-α and IFN-γ *in vitro* [34], [35].

Importantly, age- and sex-matched gnotobiotic IL-10^−/−^ mice infected with a commensal *E. coli* strain (isolated from the colonic lumen of a wildtype mouse harboring a conventional gut microbiota and lacking any relevant pathogenicity factors) did not display any disease symptoms. Thus, the induced acute enterocolitis was pathogen specific and not due to infection with any (Gram-negative) bacterial species. Interestingly, acute enterocolitis was accompanied by translocation of live *C. jejuni* into mesenteric lymph nodes of all infected animals whereas *E. coli* virtually did not spread to extraintestinal compartments.

IL-10^−/−^ mice have been used to study *C. jejuni* colonization and enteritis before. Bell *et al.* showed that *C. jejuni* induced a distinct inflammatory response in IL-10^−/−^ mice [36]. Whereas Mansfield and colleagues demonstrated that enteritis developed after more than 28 days upon *C. jejuni* infection in IL-10^−/−^ mice housed under specific pathogen free conditions (and depending on their genetic background) [37], [38], in our study, *C. jejuni* induced a rapid ulcerative colitis in gnotobiotic IL-10^−/−^ mice. Using germfree IL-10^−/−^ NF-κB^EGFP^ mice raised under isolator-conditions, Lippert *et al.* highlighted the role of NF-κB in *C. jejuni* induced pathogenesis [39]. Following infection, germfree IL-10^−/−^ NF-κB^EGFP^ mice displayed mild symptoms at day 5 and ulcerative colitis at day 14 following *C. jejuni* strain 81–176 infection. In our *C. jejuni* infected gnotobiotic IL-10^−/−^ mice acute enterocolitis had developed earlier (between day 4 and 7 p.i.) which might be due to differences in the *C. jejuni* strains used and due to the fact that our gnotobiotic animals exhibited a functional NF-kB pathway. Furthermore, germfree mice raised and housed under isolator conditions display an abnormally developed gut-associated lymphoid tissue [20], [21] and, hence, might not represent a suitable model for human *C. jejuni* infection [22].

Toll like receptors comprize essential signaling pathways involved in innate and adaptive host responses to pathogenic infections [27], but detailed *in vivo* studies on the interplay of TLRs with *C. jejuni* in the intestinal tract are scarce. In gnotobiotic mice generated by antibiotic treatment we showed previously that detection of *C. jejuni* lipooligoaccharide and CpG-DNA by host TLR-4 and TLR-9, respectively, was essential for mediating pro-inflammatory immune responses upon *C. jejuni* infection [22]. Clinical and histopathological signs of inflammation, however, were rather subtle in these mice. In the presented study, we could clearly demonstrate that signaling of bacterial lipooligoaccharide and, less distinctly, lipoproteins by TLR-4 and -2, respectively, was essentially involved in mediating acute enterocolitis in infected gnotobiotic IL-10 deficient mice. This is well in line with *in vitro* studies showing that *C. jejuni* activates TLR-2 and TLR-4 in different human, murine and avian cell lines [28], [29], [30], [31], [32]. Whereas following *C. jejuni* infection IL-10^−/−^ mice lacking TLR-4 were significantly less compromized as compared to IL-10^−/−^ controls, a not significant, but clear trend towards a better clinical outcome and less distinct histopathological changes seen in the colonic mucosa could be observed in the heterogenous cohort of TLR-2 IL-10 double deficient animals. In both, IL-10^−/−^ mice lacking TLR-2 or TLR-4, amelioration of inflammation was associated with significantly lower numbers of apoptotic cells and T lymphocytes in the colon *in situ*. These diminished cellular responses were accompanied by lower secretion of pro-inflammatory cytokines such as TNF-α, IFN-γ and IL-6 in *ex vivo* colonic biopsies derived from *C. jejuni* infected TLR-4 IL-10 double deficient mice. In addition, increases in Tregs were less distinct in the colon of TLR deficient animals further indicating that TLRs -2 and -4 play essential roles in initiating inflammation by pro-inflammatory signals.

Furthermore, virtually no translocation of live *C. jejuni* to mesenteric lymphnodes in infected TLR-4 IL-10 double deficient, but not IL-10^−/−^ mice indicates an uncompromized intestinal epithelial barrier function in the former. The immunopathological impact of TLR-4 dependent signaling of lipooligosaccharide in human campylobacteriosis is even more underlined by the fact that humans are up to 1000 times more sensitive to TLR-4 ligands than rodents [40]. Taken together, we here present a novel *C. jejuni* infection model mimicking acute enterocolitis in human campylobacteriosis. The course of disease mimics the situation seen in immunocompromized humans. Furthermore, the observed non self-limited acute ulcerative colitis following *C. jejuni* infection of gnotobiotic IL-10 deficient mice underlines the crucial role of the host specific microbiota in protecting against invading pathogens and underlines the importance of the anti-inflammatory cytokine IL-10 in *C. jejuni* induced immunopathology.

We conclude that TLR-4-, and to a lesser extent TLR-2-, signaling play important roles in mediating *C. jejuni* induced acute enterocolitis. The presented study provides valuable tools to gain further insights into the immunological and molecular interplays between *C. jejuni* and innate immunity in human campylobacteriosis.

## Materials and Methods

### Ethics Statement

Animal experiments were conducted according to the European Guidelines for animal welfare (2010/63/EU) with approval of the commission for animal experiments headed by the “Landesamt für Gesundheit und Soziales” (LaGeSo, Berlin, Germany; Registration numbers: G0173/07 and G135/10). Animal welfare was monitored twice daily by assessment of clinical conditions.

### Mice

IL-10^−/−^ mice (in C57BL/10 background, B10) were bred and maintained in the facilities of the “Forschungsinstitut für Experimentelle Medizin” (FEM, Charité - Universitätsmedizin, Berlin, Germany), under specific pathogen-free (SPF) conditions. In order to gain double-deficient animals, TLR-2^−/−^ and TLR-4^−/−^ mice (in B10 background each) were crossed to IL-10^−/−^ mice and backcrossed at least 7 generations before use.

To eradicate the commensal gut flora, mice were transferred to sterile cages and treated by adding ampicillin (1 g/L; Ratiopharm), vancomycin (500 mg/L; Cell Pharm), ciprofloxacin (200 mg/L; Bayer Vital), imipenem (250 mg/L; MSD), and metronidazole (1 g/L; Fresenius) to the drinking water ad libitum as described earlier [41] starting at 3 weeks of age right after weaning. Age matched female mice were subjected to the quintuple antibiotic treatment for 4 months before the infection experiment.

### Bacterial Strains

The *E. coli* strain used is a commensal isolate derived from our conventionally colonized, naive C57BL/6j wildtype mice and identical with *E. coli* M described earlier [42]. The genome does not contain known virulence factors of pathogenic *E. coli* such as stx, 1 and 2, catA, hlyA, cspA, katP and astA. This was confirmed in a reference laboratory by PCR analysis. The *C. jejuni* strain B2 was kindly provided by Prof. Dr. Uwe Groß, University of Göttingen (Germany). This strain was isolated from a patient suffering from bloody diarrhea, has shown to be invasive *in vitro*, and its main properties have been described in detail by Tareen et al. [33].

### *C. jejuni* and *E. coli* Infection of Mice

Two days prior to infection the antibiotic cocktail was withdrawn and changed to sterilized tap water. Right before oral infection, sterility of each mouse was confirmed by transferring individual fecal samples to thioglycollate enrichment broths. Then, mice were infected with approximately 10^9^ viable CFU of *C. jejuni* strains B2 by gavage in a total volume of 0.3 mL PBS on two consecutive days as described earlier in detail [22]. For infection with a Gram-negative control species, live *E. coli* were isolated from naïve, healthy 3 months old C57BL/10 mice by culture on MacConkey media. This commensal *E. coli* strain was subcultivated on blood agar and mice with approximately 10^9^ viable CFU of *E. coli* by gavage in a total volume of 0.3 mL PBS on two consecutive days in parallel to the respective *C. jejuni* B2 groups.

### Clinical Score

To assess clinical signs of *C. jejuni* induced infection on a daily basis, a standardized cumulative clinical score (maximum 12 points; modified according to [43] addressing the occurrence of blood in feces (0 points: no blood; 2 points: microscopic detection of blood by the Guajac method using Haemoccult, Beckman Coulter/PCD, Krefeld, Germany; 4 points: overt blood visible), diarrhea (0: formed feces; 2: pasty feces; 4: liquid feces), and the clinical aspect (0: normal; 2: ruffled fur, less locomotion; 4: isolation, severely compromized locomotion, pre-final aspect) was used.

### Sampling Procedures, Determination of Colon Length and Histopathology

Mice were sacrificed by isofluran treatment (Abbott, Germany). From each mouse samples derived from the gastrointestinal tract (stomach, duodenum, ileum, colon) were collected in parallel for histological, microbiological and immunobiological analyses. Cardiac blood and tissue samples from mesenteric lymphnodes, spleen, liver and the gastrointestinal tract were asserved under sterile conditions. Histopathological changes were determined in colon samples immediately fixed in 5% formalin and embedded in paraffin. Sections (5 µm) were stained with respective antibodies for immunohistochemistry.

### Immunohistochemistry

*In situ* immunohistochemical analysis of colonic paraffin sections were performed as described previously [22], [44], [45]. Primary antibodies against cleaved caspase-3 (Asp175, Cell Signaling, USA, 1∶200), CD3 (#N1580, Dako, Denmark, dilution 1∶10), FOXP-3 (FJK-16s, eBioscience, 1∶100), and B220 (eBioscience, San Diego, CA, USA, 1∶200) were used. For each animal, the average number of positive stained cells within at least six independent high power fields (HPF, 400x magnification) were determined microscopically and subjected to statistical analysis as indicated.

### Cytokine Detection in Colon Culture Supernatants

Colon biopsies were cut longitudinally, washed in PBS and strips of 1 cm^2^ placed in 24-flat-bottom well culture plates (Nunc, Wiesbaden, Germany) containing 500 µL serum-free RPMI 1640 medium supplemented with penicillin (100 U/mL) and streptomycin (100 µg/mL; PAA Laboratories). After 18 h at 37°C, culture supernatants were tested for TNF-α, IFN-γ, MCP-1 and IL-6 by the Mouse Inflammation Cytometric Bead Array (CBA; BD Biosciences) on a BD FACSCanto II flow cytometer (BD Biosciences).

### Microbiota Analyses

Cultural analyses, biochemical identification and molecular detection of luminal bacterial communities from (stomach, duodenum, ileum, and) colon as well as feces were performed as previously described [22], [41], [42], [44]. For bacterial translocation experiments, mesenteric lymphnodes draining the small intestine, spleen, liver (1 cm^2^) and cardiac blood (1 mL) were transferred into thioglycollate enrichment broths under sterile conditions and cultivated for 7 days. Turbid broths were streaked onto sheep blood, MacConkey as well as Karmali agar in order to detect translocated live *E. coli* and *C. jejuni* followed by species identification via biochemical and molecular methods [22]. Given that bacterial growth in enrichment broths cannot be quantitated, the relative rates of translocating *C. jejuni* or *E. coli* are expressed in %.

### Statistical Analysis

Mean values, medians, standard deviations, and levels of significance were determined using appropriate tests as indicated (two-tailed Student’s *t*-Test, Mann-Whitney-U Test). Two-sided probability (*P*) values ≤0.05 were considered significant. All experiments were repeated at least twice.
